# Supplementary material for: Proteomics Analysis of the Polyomavirus DNA Replication Initiation Complex Reveals Novel Functional Phosphorylated Residues and Associated Proteins
Source: Int J Mol Sci. 2024 Apr 21;25(8):4540. doi: 10.3390/ijms25084540 (PMC11049971; doi:10.3390/ijms25084540)
Supplement: Supplementary file 1 [file ijms-25-04540-s001.zip › ijms-2946242-supplementary/02 Supplementary Data - PyV Bioinfo-Final/02 Supplementary Data Files- PyV Bioinfo/Supplementary Figures -IJMS format-1.pdf]

Supplementary Figures

Proteomics analysis reveals novel phosphorylated residues and associated proteins of the polyomavirus DNA replication initiation complex.

Rama Dey-Rao1*, Shichen Shen 2, Jun Qu 2, Thomas Melendy1

[1] Department of Microbiology & Immunology, Jacobs School of Medicine & Biomedical Sciences, University at Buffalo, Buffalo, NY, United States of America
[2] Department of Pharmaceutical Sciences, University at Buffalo, State University of New York at Buffalo, Buffalo, NY, United States of America
* Correspondence: dey@buffalo.edu

A

B

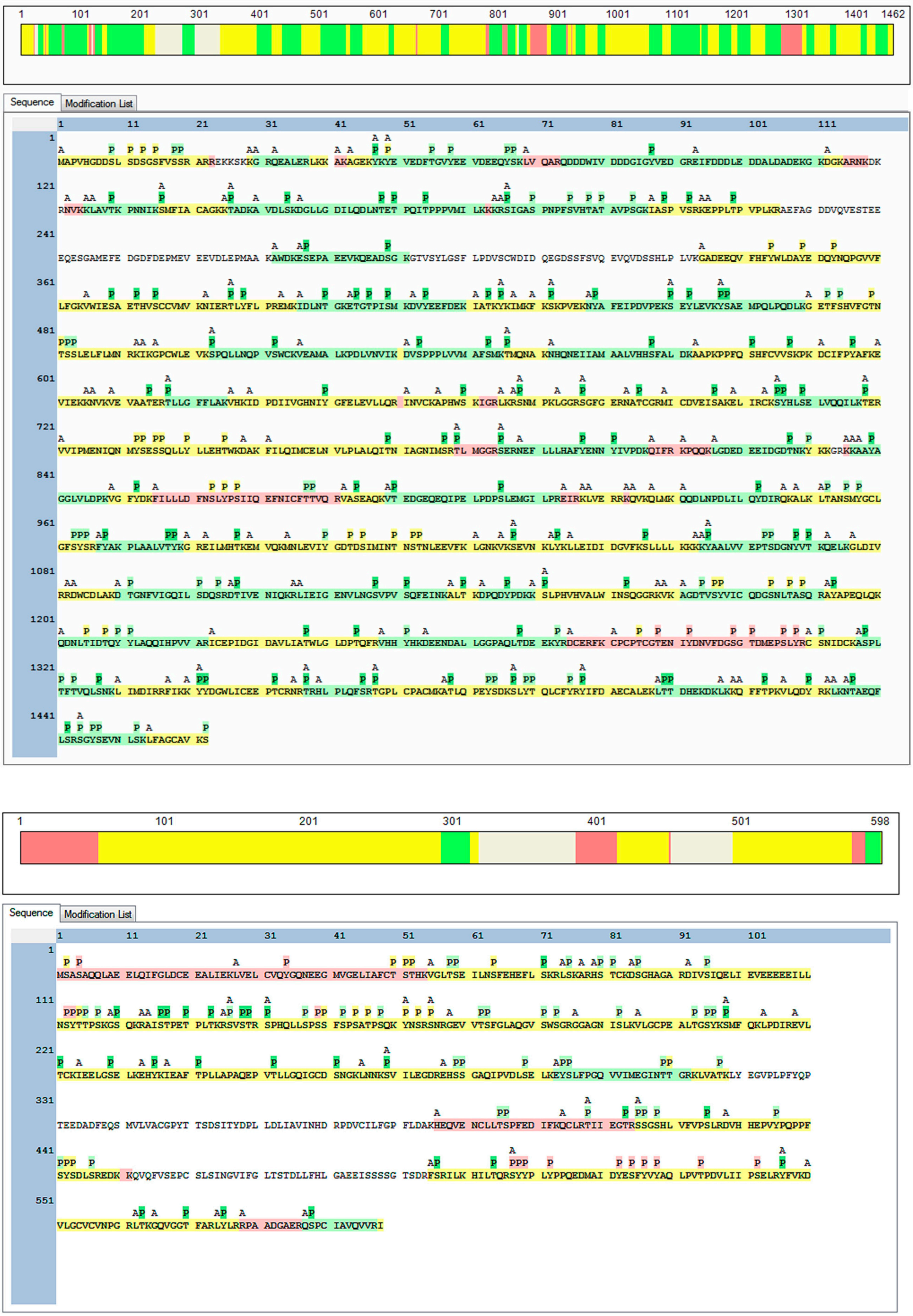
1 101 201 301 401 501 601 701 801 901 1001 1101 1201 1301 1401 1462
Sequence
Modification List
1 101 201 301 401 501 598
Sequence
Modification List

C

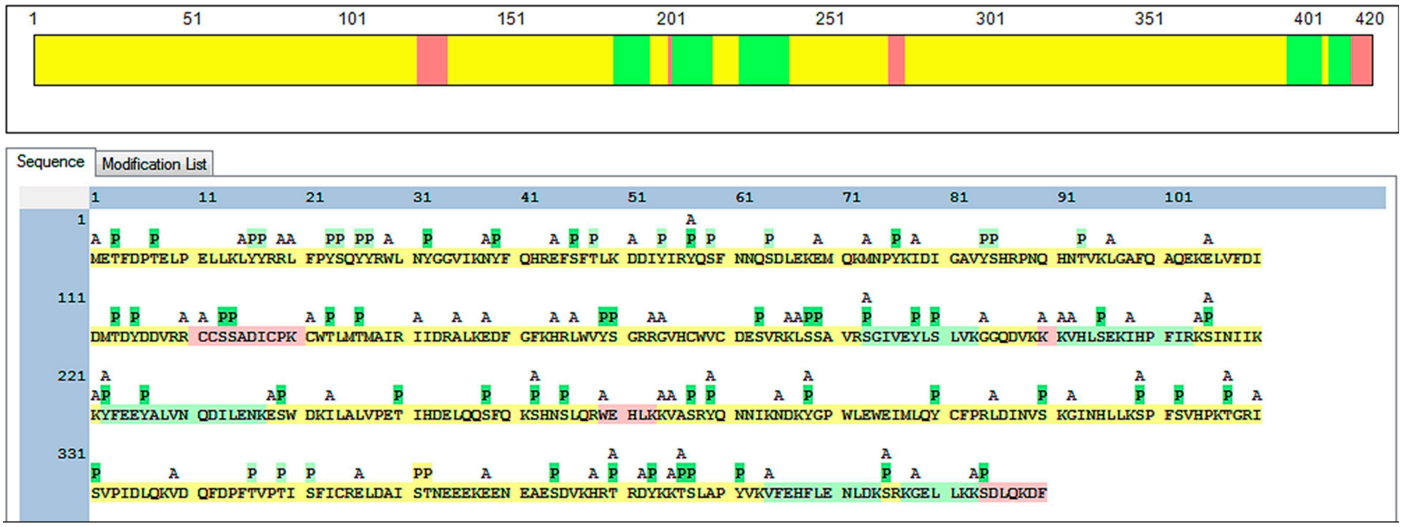

D

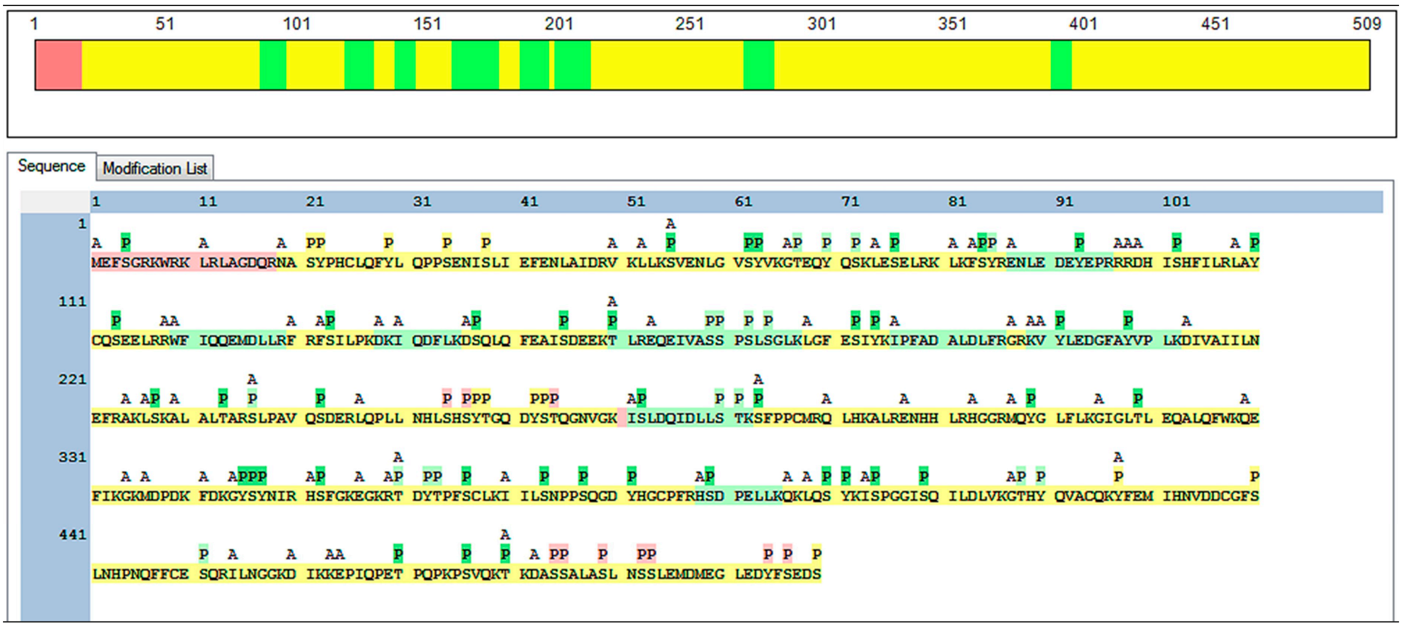

Figure S1 A-D: PAARs on Polprim-complex by MS analysis

MS2 analysis of the IP eluates resulted in very similar sequence maps (modification list) from both Cont and ETO treatments in each of the four subunits of Polprim complex. Therefore, only one representative from each is demonstrated. Sequence coverage of detected tryptic peptides was 93% for (A) Catalytic subunit p180 (POLA1), 82% for (B) Regulatory subunit p68 (POLA2), and 100% for both (C) Enzymatic subunit p48 (PRIM1) and (D) Enzymatic subunit p58 (PRIM2) respectively. Some peptides (R23-K28, R225-K271 and K291-K333 in POLA1 as well as K318-K385 and K451- R494 in POLA2) remained undetected. For further details of PAARs on Polprim subunits see Table S3. The overall detection coverage (colored by confidence levels of peptide detection) by MS/MS analysis is shown by a scale bar graphic on top of the full sequence map. The identified tryptic peptides and phosphorylation (P) are colored by confidence of identification: green (high) yellow (medium) red (low) and white (undetected by MS/MS). The acetylation of free amine groups is on any aa including Lys (K) and Arg (R) termini and occur spontaneously during experimental steps and may not be biologically meaningful unless validated. To avoid overinterpretation of the data, we do not include acetylated residues in our findings. Abbreviations: P= phosphorylated residues, A= acetylated residues.

A

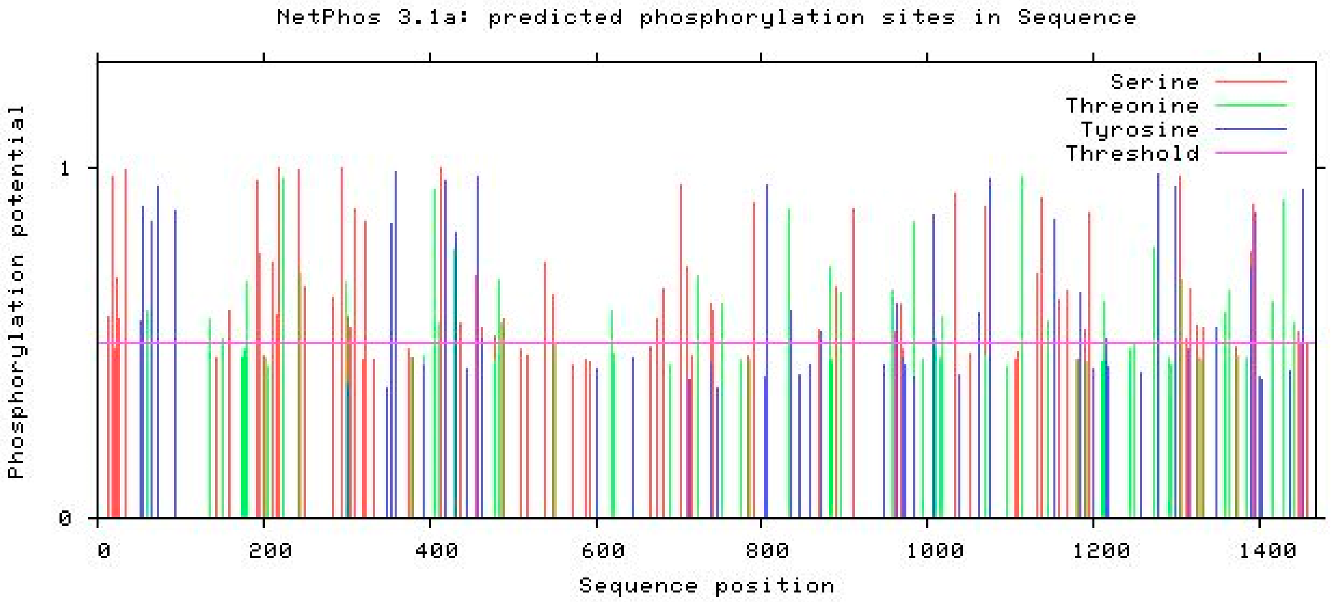

NetPhos 3.1a: predicted phosphorylation sites in Sequence
Serine
Threonine
Tyrosine
Threshold
Phosphorylation potential
1
0
0
200
400
600
800
1000
1200
1400
Sequence position

B

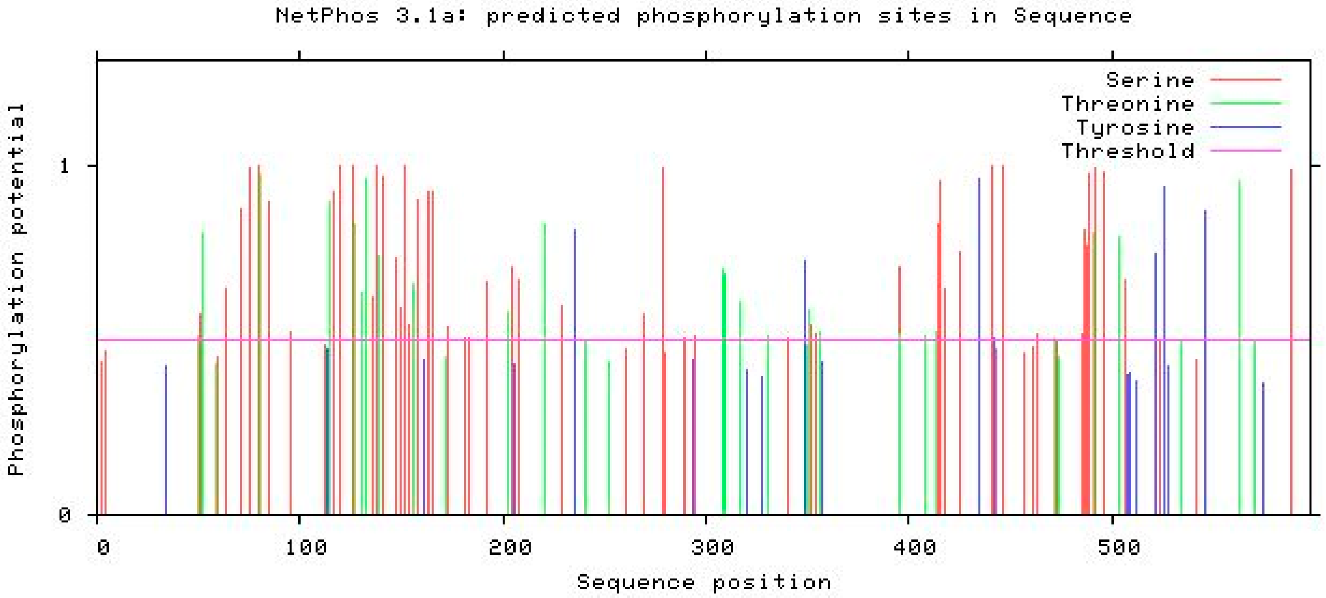

NetPhos 3.1a: predicted phosphorylation sites in Sequence
Serine
Threonine
Tyrosine
Threshold
Phosphorylation potential
1
0
0
100
200
300
400
500
Sequence position

C

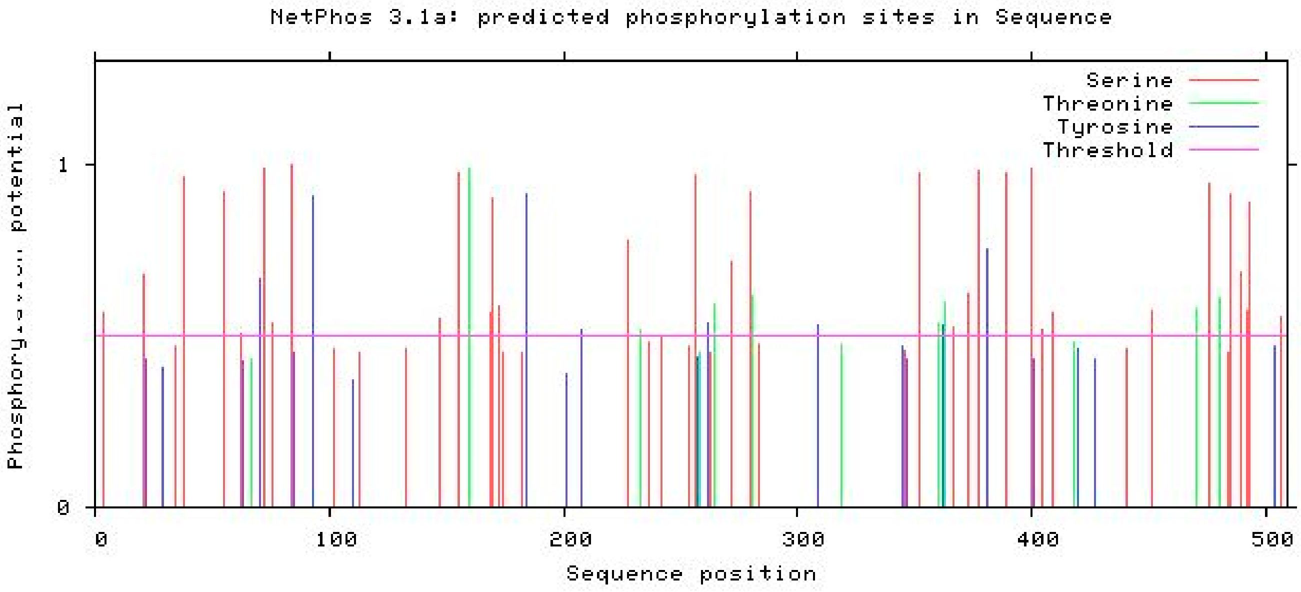

D

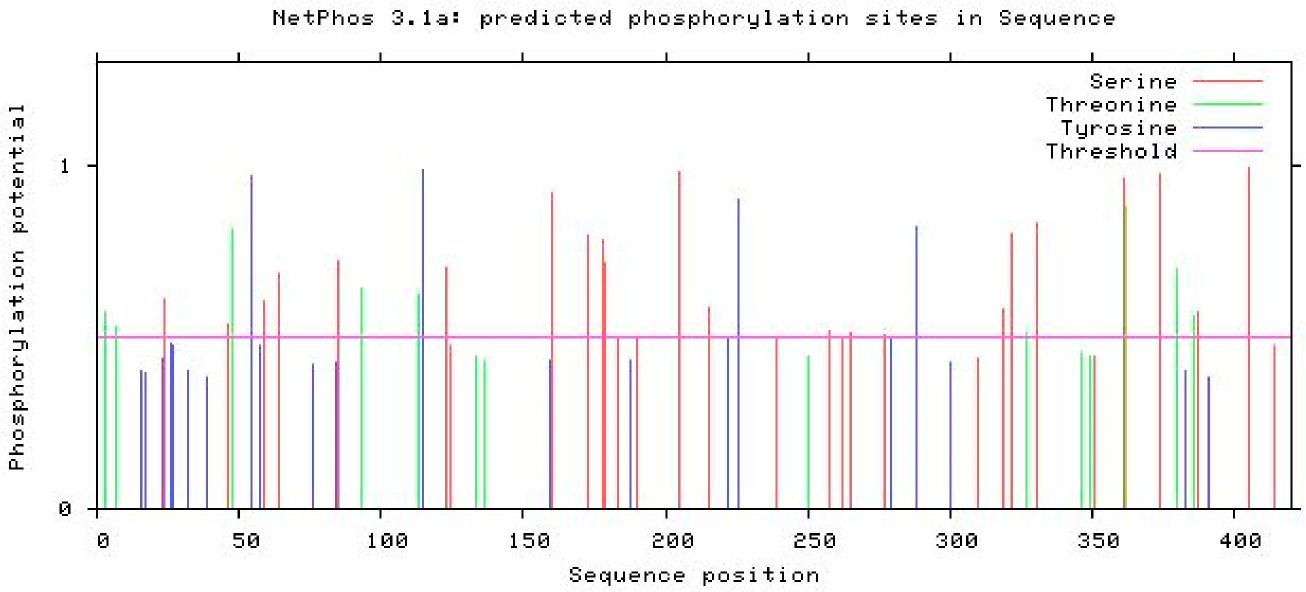

Figure S2 A-D: *In silico* predictions of PAARs on Polprim-complex.

Graphical output of *in silico* PAAR predictions via Netphos v3.1: (A) POLA1 (p180, 1462 aa), (B) POLA2 (p68, 598 aa) (C) PRIM1 (p48, 420 aa), and (D) PRIM2 (p58, 509 aa) with 123, 81, 35 and 48 predicted phospho- S, T, Ys predicted in the four subunits respectively (More details can be found in Supplementary Table 3, sheet 5). X-axis is the number of aa residues. Y-axis is a measure of the phosphorylation potential on a scale of 0 to >1 with a cut off at ≥0.5 (magenta line) for positive predictions.

A

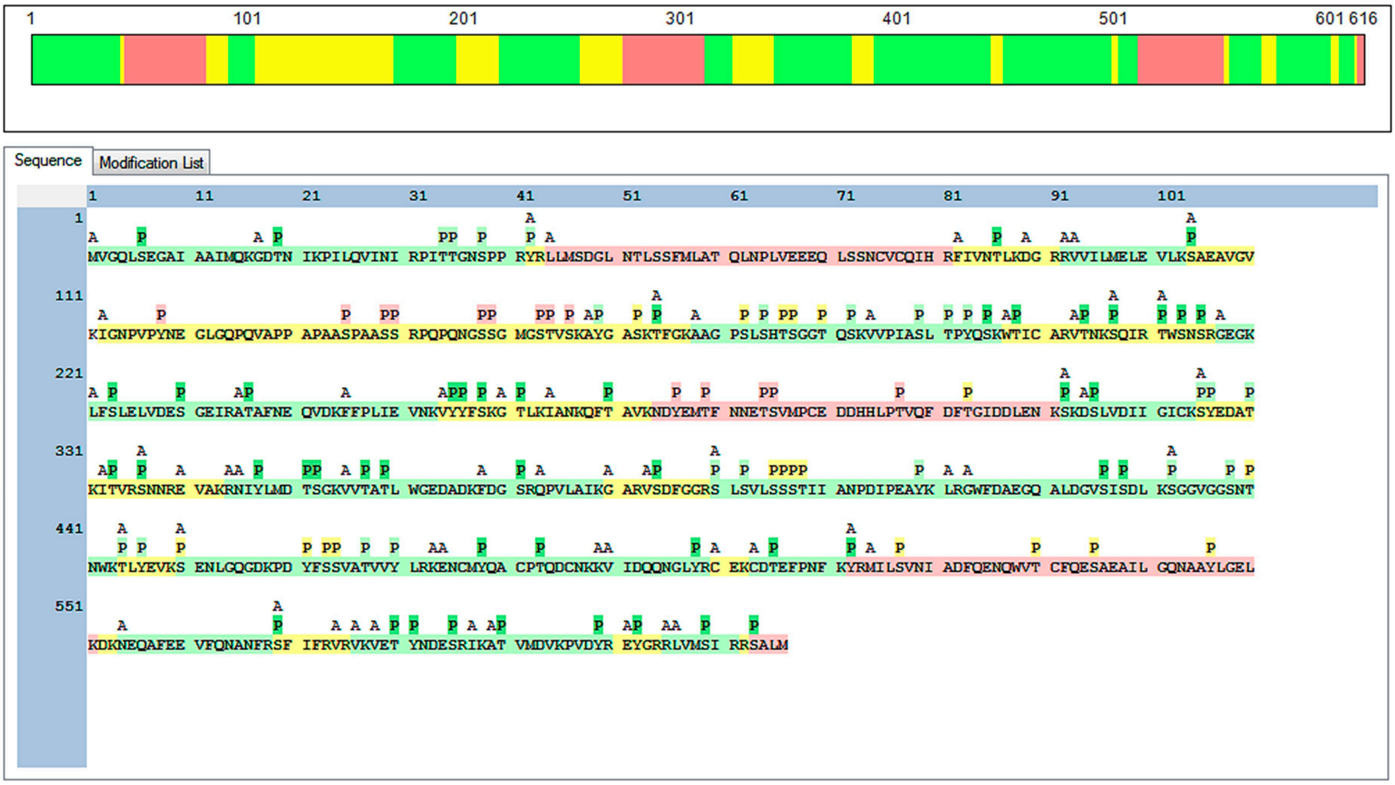

B

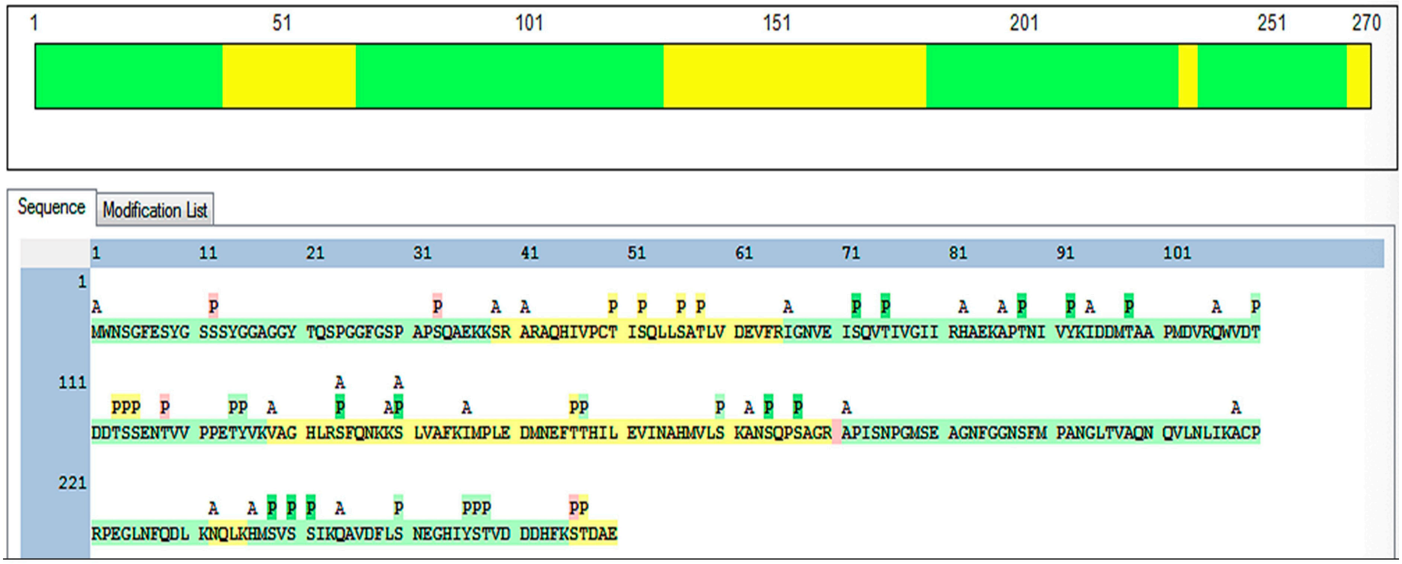

C

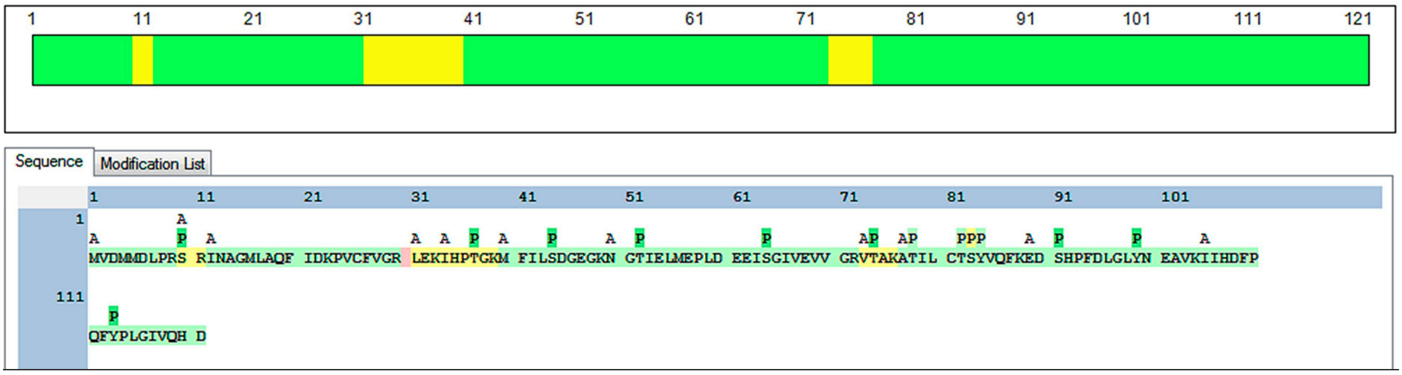

Figure S3 A-C: PAARs on RPA-complex by MS analysis

LC-MS/MS analysis of the IP eluates resulted in very similar sequence maps (with modifications) from both Cont. and ETO treatments in each of the three subunits of RPA-complex. Therefore, like SV40 LT and Polprim only one representative from each is demonstrated. Sequence coverage of detected tryptic peptides in the entire protein is 100% for all three subunits- (A) RPA1 (p70), (B) RPA2 (p32) and (C) RPA3 (p14). For further details of PAARs on RPA subunits see Table S5. The overall detection coverage (colored by confidence levels of peptide detection) by MS/MS analysis is shown by a scale bar graphic on top of the full sequence map. The identified tryptic peptides and phosphorylation (P) are colored by confidence of identification: green (high) yellow (medium) red (low) and white (undetected by MS/MS). The acetylation of free amine groups is on any aa including Lys (K) and Arg (R) termini and occur spontaneously during experimental steps and may not be biologically meaningful unless validated. To avoid overinterpretation of the data, we do not include acetylated residues in our findings. Abbreviations: P= phosphorylated residues, A= acetylated residues.

A

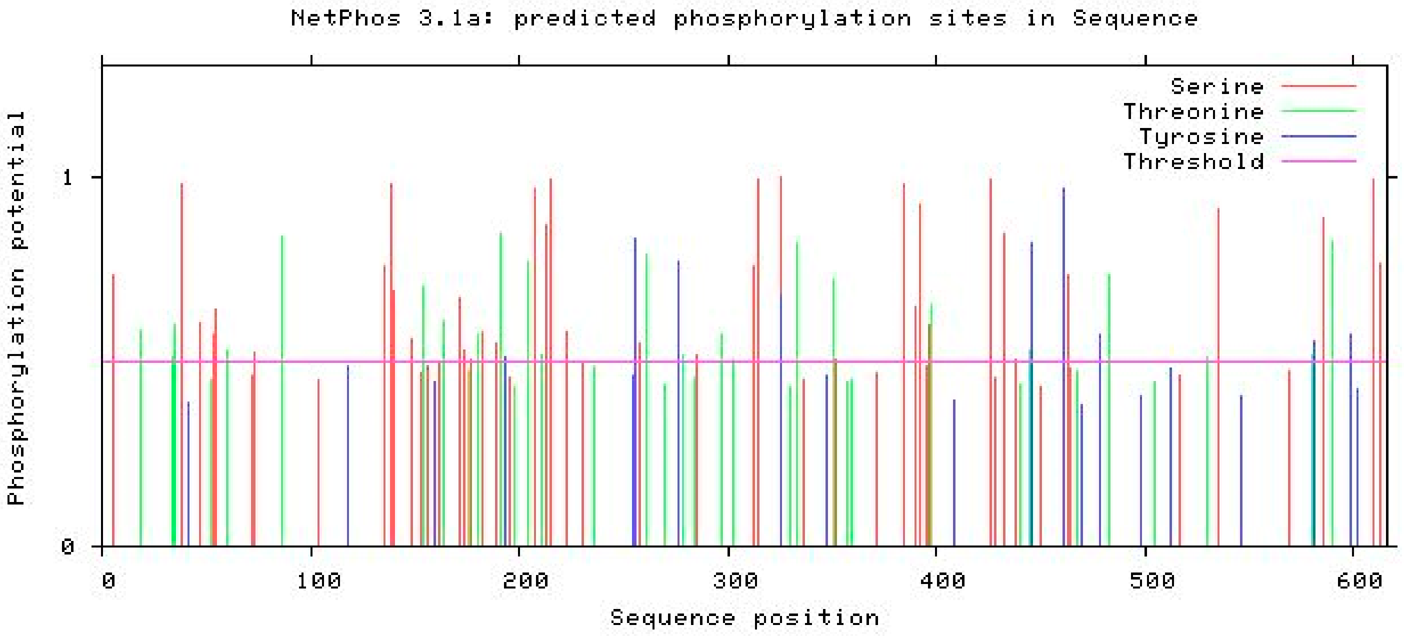

NetPhos 3.1a: predicted phosphorylation sites in Sequence
Serine
Threonine
Tyrosine
Threshold
Phosphorylation potential
Sequence position

B

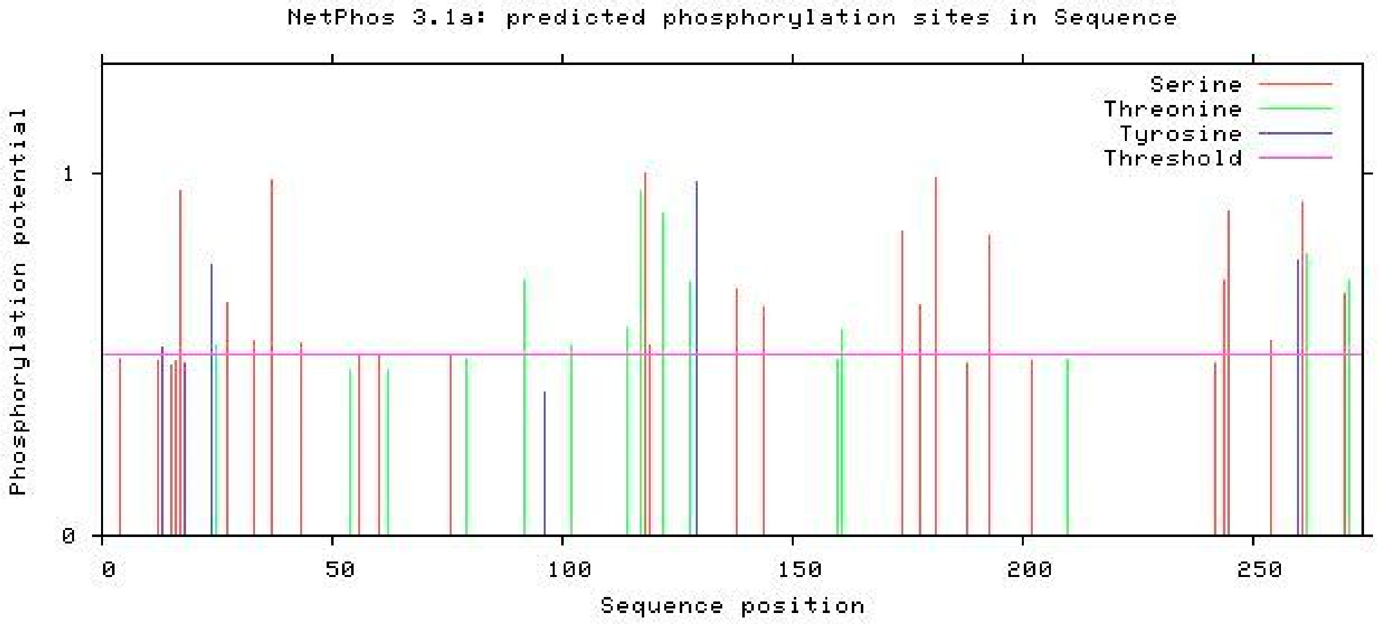

NetPhos 3.1a: predicted phosphorylation sites in Sequence
Serine
Threonine
Tyrosine
Threshold
Phosphorylation potential
Sequence position

C

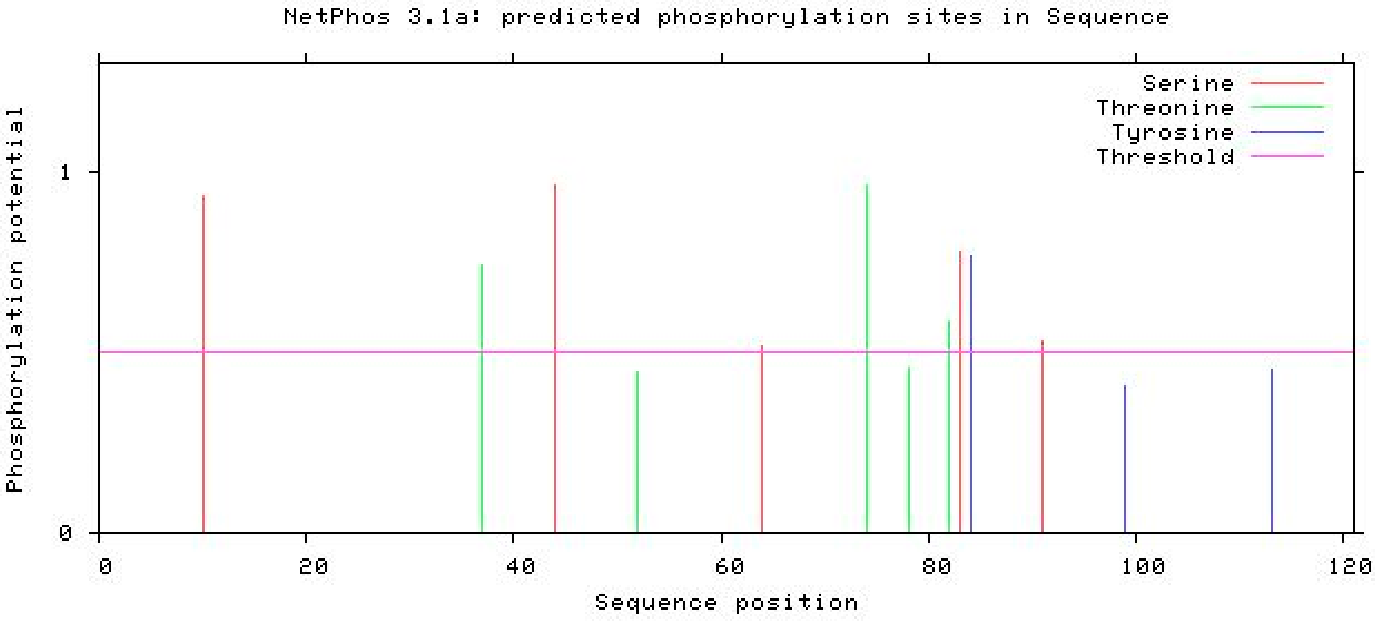

Figure S4 A-C: *In silico* predictions of PAARs on RPA-complex.
Graphical output of *in silico* predictions via Netphos v3.1 in: (A) RPA1 (p70, 616 aa), (B) RPA2 (p32, 270 aa) and (C) RPA3 (p14, 121 aa) with 70, 34 and 9 predicted phospho- S, T, and Ys on the three subunits, respectively. (See further details in Supplementary Table 5, sheet 4). X-axis is the number of aa residues. Y-axis is a measure of the phosphorylation potential on a scale of 0 to >1 with a cut off at ≥0.5 (magenta line) for positive predictions.

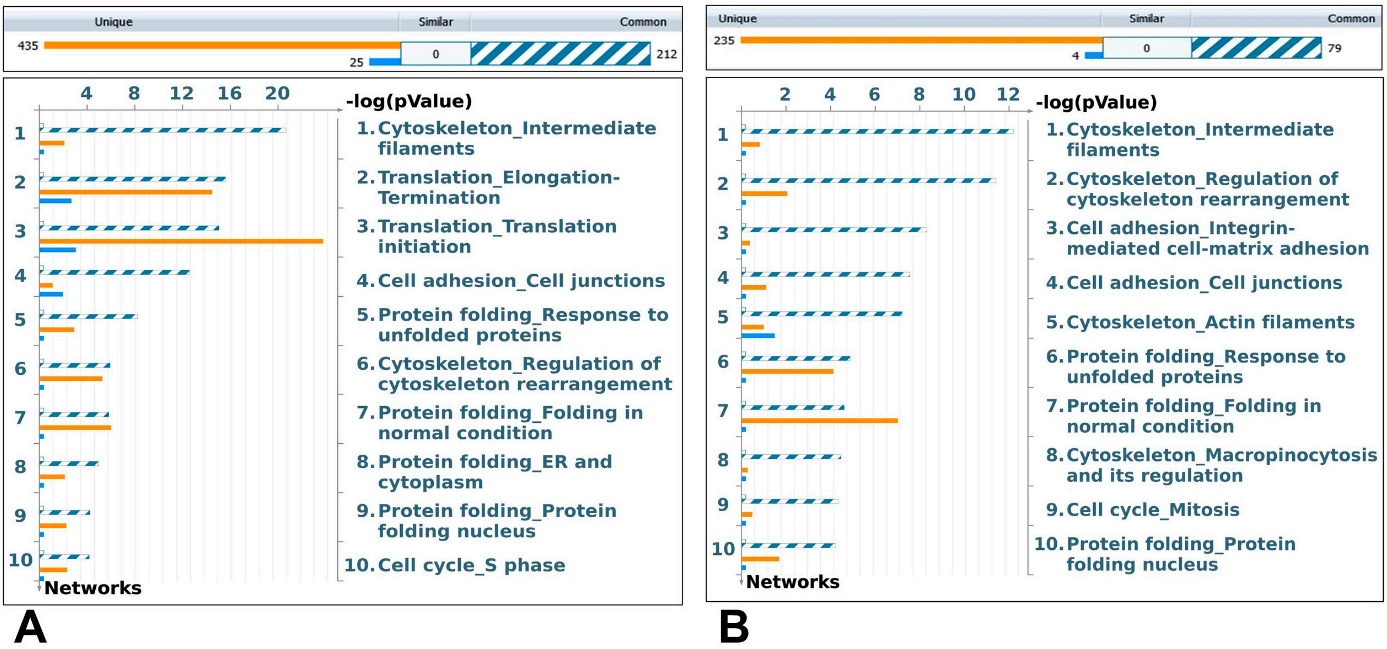

Figure S5 A-B: GO Enrichment Analysis; Process Networks wih Polprim and RPA associated proteins

Gene content was aligned between the individual uploaded lists of associated proteins with Polprim and RPA within the web-based integrative software suite (MetaCore). The genes sorted into three categories as seen in the histograms: 1) common to both Cont and ETO (hatched blue and white), 2) unique to Cont (solid orange) and 3) unique to ETO (solid blue) in the top panels of: (**A**) Polprim-associated gene IDs split into 212 common, 435 and 25 objects unique to Control and ETO respectively and (**B**) RPA associated gene IDs sorted to 79 common, 235 and 4 unique NOs to Control and ETO respectively. The top 10 enriched process networks are arranged in negative log p-values from most to least relevant in the bottom panels of the GO enrichment analyses for protein lists from (A) Polprim-complex and (B) RPA-complex. Sorting is done for p-value of the "common" set of genes/NOs. " See Table 7 -sheet 2-3 for underlying statistics. Translation related networks were the most enriched networks due to the coIP of several ribosomal proteins (RP) as integral components of the ribosome that interact with RNA and other cellular components with high affinity. RP aggregates are often found in IPs from lysates of robustly growing cells or in stress response.

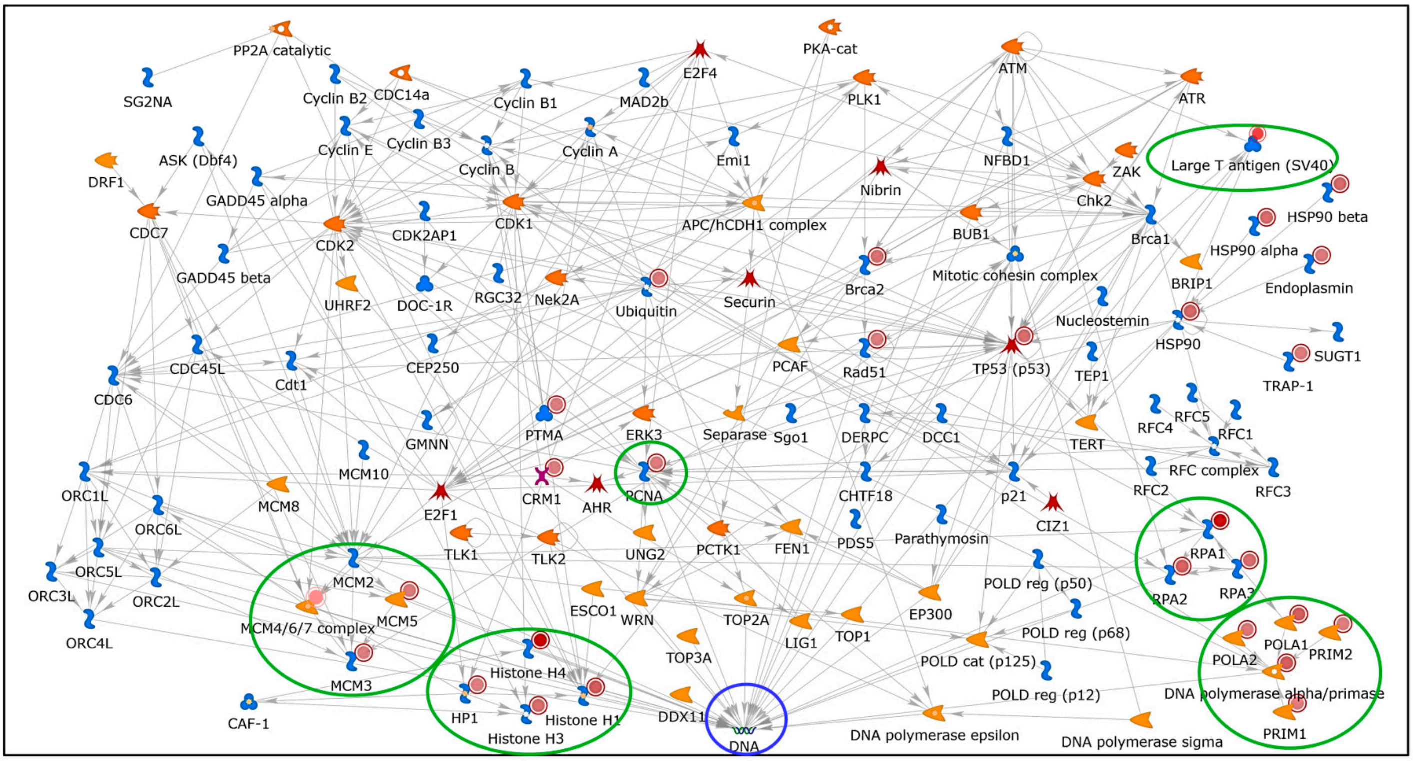
PP2A catalytic
PKA-cat
SG2NA
Cyclin B2
CDC14a
Cyclin B1
MAD2b
E2F4
PLK1
ATM
ATR
ASK (Dbf4)
Cyclin E
Cyclin B3
Cyclin B
Cyclin A
Emi1
NFBD1
Nibrin
ZAK
Large T antigen (SV40)
DRF1
GADD45 alpha
CDK1
APC/hCDH1 complex
Chk2
BUB1
Brca1
HSP90 beta
CDC7
CDK2
CDK2AP1
HSP90 alpha
GADD45 beta
Mitotic cohesin complex
BRIP1
Endoplasmin
UHRF2
DOC-1R
RGC32
Nek2A
Ubiquitin
Securin
Brca2
Nucleostemin
HSP90
SUGT1
CDC45L
Cdt1
CEP250
PCAF
Rad51
TP53 (p53)
TEP1
TRAP-1
CDC6
PTMA
ERK3
Separase
Sgo1
DERPC
DCC1
RFC4
RFC5
RFC1
GMNN
TERT
RFC complex
MCM10
CRM1
AHR
PCNA
CHTF18
p21
RFC2
RFC3
ORC1L
MCM8
E2F1
CIZ1
ORC6L
PDS5
Parathymosin
TLK1
TLK2
UNG2
PCTK1
FEN1
RPA1
ORC5L
MCM2
POLD reg (p50)
ORC3L
ORC2L
MCM5
ESCO1
WRN
TOP2A
EP300
RPA2
RPA3
MCM4/6/7 complex
LIG1
TOP1
POLD reg (p68)
POLA1
ORC4L
TOP3A
PRIM2
Histone H4
POLD cat (p125)
POLA2
MCM3
POLD reg (p12)
DNA polymerase alpha/primase
HP1
Histone H1
DDX11
CAF-1
Histone H3
DNA
DNA polymerase epsilon
DNA polymerase sigma
PRIM1

A

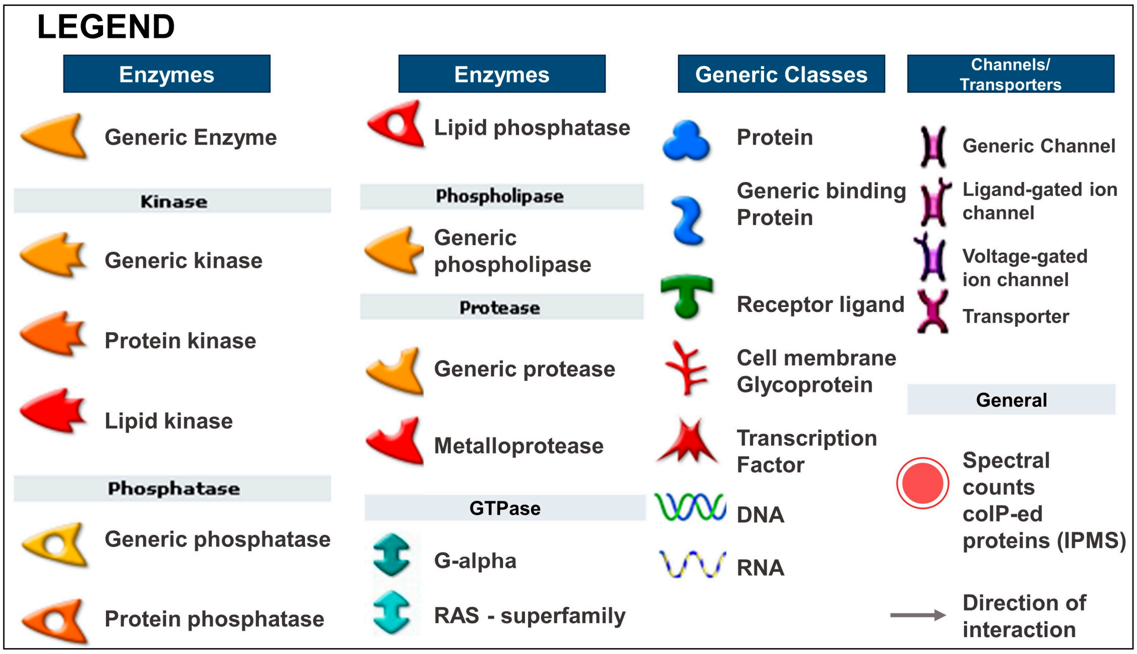
LEGEND
Enzymes
Generic Enzyme
Kinase
Generic kinase
Protein kinase
Lipid kinase
Phosphatase
Generic phosphatase
Protein phosphatase
Enzymes
Lipid phosphatase
Phospholipase
Generic phospholipase
Protease
Generic protease
Metalloprotease
GTPase
G-alpha
RAS - superfamily
Generic Classes
Protein
Generic binding Protein
Receptor ligand
Cell membrane Glycoprotein
Transcription Factor
DNA
RNA
Channels/ Transporters
Generic Channel
Ligand-gated ion channel
Voltage-gated ion channel
Transporter
General
Spectral counts coIP-ed proteins (IPMS)
Direction of interaction

#	Process	%	p-Value	Genes from Active Data
1	DNA metabolic process	64.38	1.151E-92	RAD51, POLA2
2	DNA replication	43.84	2.096E-90	PRIM2, MCM3, MCM4, RPA1, PCNA, POLA1, RPA3, MCM5, MCM7, PRIM1, RPA2, POLA2
3	Cell cycle	67.81	2.111E-81	MCM3, MCM4, TP53, MCM5, MCM7, BRCA2
4	DNA-templated DNA replication	34.25	8.108E-74	RPA1
5	Chromosome organization	56.16	1.418E-67	RPA1, TP53, BRCA2
6	Cellular response to DNA damage stimulus	51.37	5.978E-67	RPA1, PCNA, RAD51, TP53, RPA3, MCM7, BRCA2, RPA2
7	DNA repair	41.78	1.043E-58	RPA1, PCNA, POLA1, RAD51, RPA3, BRCA2, RPA2
8	cellular response to stress	60.96	8.806E-57	TP53

B

Figure S6 A-B: Cell Cycle _ S Phase related network and biological processes

(A) Cell Cycle_S Phase was the top overconnected network associated with the term "DNA replication" in the MetaCore database. The final merged network combines the prebuilt network in the database with our activated experimental dataset, along with the addition of SV40-LT (indicated using a generic protein descriptor). Twenty-six gene IDs from our IPMS dataset: LT, PCNA, MCM3, MCM4, MCM5, MCM7, TP53, Rad51, CRM1(XPO1), BRCA2, Ubiquitin, HSP90 family: HSP90 alpha (HSP90AA1), HSP90 beta (HSP90B1), PTMA, TRAP1 and members of the Histone family, subunits of the Polprim and RPA complexes, were included in this network (solid red circles). Several proteins (green ovals). within the network were also previously reported to be associated with the SV40 DNA replication fork (Table S8 sheet 3). Individual proteins or objects are represented as nodes and different shapes of the nodes represent functional classes of proteins and are clarified in the legend. (Statistics: Table S9, sheets 1-9). While the enrichments and networks building statistics are calculated according to our uploaded experimental dataset (gene IDs) without spectral counts (SC), the SC are used to set thresholds to allow visualization of the semi-quantitative measure for coIP-ed protein abundance in the IP eluates or cell lysate (solid red circles associated with network object). The network of interactions (also called edges: grey arrows) between proteins (with direction) were based on the curated knowledgebase within Metacore. The arrowheads indicate the direction of the interactions. Large T-antigen was the only SV40 specific protein added to every network analysis. It mapped to a generic protein in Metacore, to which the red solid circle was added, the color consistent with spectral counts in our MS results.

(B) The significant biological processes enriched within the network were related to DNA metabolic process, DNA replication, cell cycle, and DNA damage and repair, among others and included several genes from our results (Figure S7). Several proteins such as MCM3, MCM5, MCM4, MCM7 that are part of the mammalian replication machinery were found to coIP with SV40 LT and Polprim. This is not yet fully understood. Perhaps LT's association with Polprim and RPA-results in larger replication complexes that is able to precipitate the MCMs. In summation, this network validates our IPMS protein dataset to be representative of both SV40 and human DNA replication in the cell cycle. The enrichments and network building statistics were calculated according to our experimental dataset (For statistics underlying the "Cell cycle_ S phase" network see Table S9, sheets 1-9).

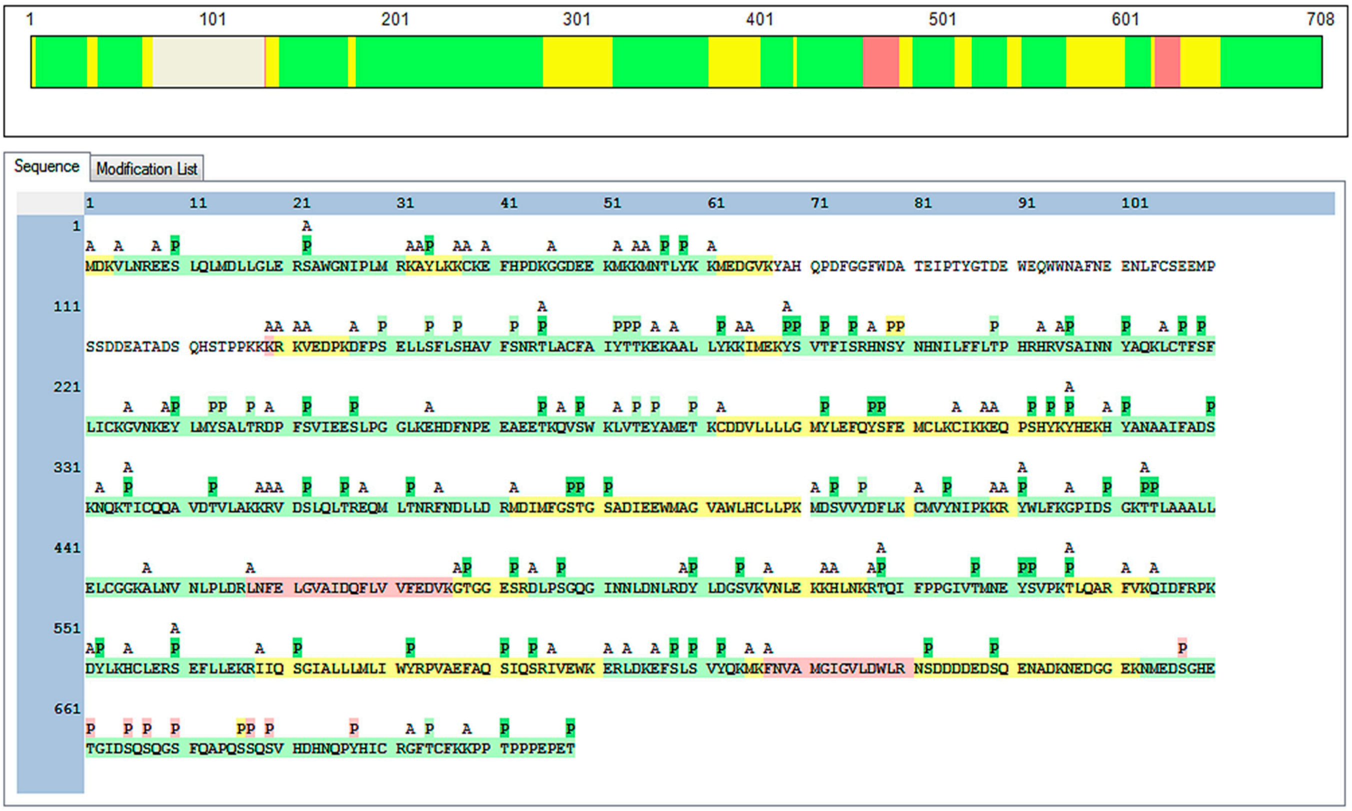

Figure S7: PAARs on SV40 LT by MS analysis

PAARs on SV40 LT amino acid sequence using MS/MS analysis. Only one representative sequence map with modifications for SV40 LT is shown since there was a large overlap between Cont and ETO. Sequence coverage of detected peptides (by MS) was 91% for both treatments. Tryptic peptide K67-K129 remained undetected by MS/MS (For further details of SV40 LT PAARs see Table S1). The tryptic peptide detection and S/T/Y phosphor-sites (P) are highlighted with colors by confidence levels (scale bar graphic on top of the full sequence map): green (high) yellow (medium) red (low) and white (undetected by MS/MS). To avoid overinterpretation of the data the spontaneous O and A modifications of free amine groups are not included in this report (see Methods). Abbreviations: P= phosphorylated residues, A= acetylated residues.

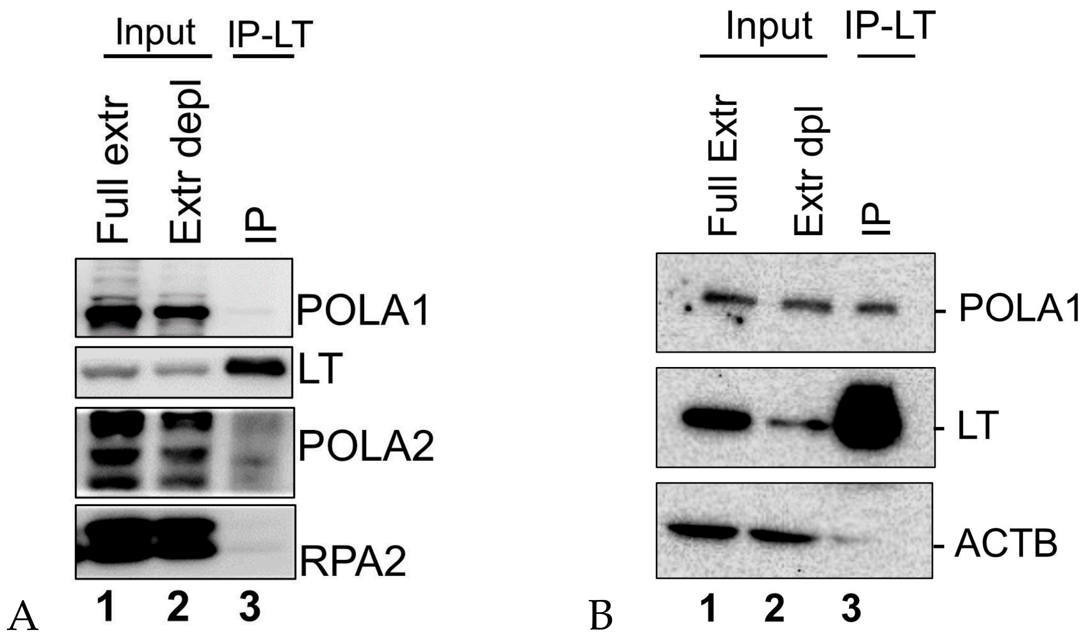

Figure S8A-B : Co-immunoprecipitation of POLA1, POLA2, RPA2 with SV40 LT

Two immunoprecipitation experiments were performed with HEK293T cell exptract (Input) , before binding (Full extr) and after binding (Extr depl)with SV40 LT-specific monoclonal antibody. A) Immunoblot of the Input (lanes 1, 2) and IP-LT (lane 3) showed POLA1, POLA2, and RPA2 co-IP with LT. B) Input (Lanes 1, 2) and IP-LT (Lane 3) showed POLA1, LT and ACTB

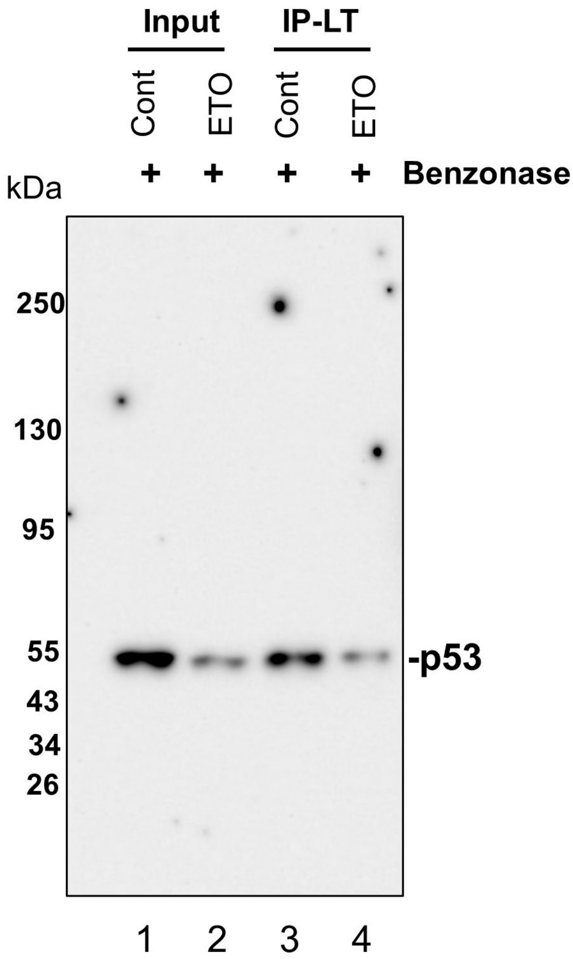

Figure S9: Co-immunoprecipitation of p53 with SV40 LT after Benzonase treatment

Immunoprecipitation experiments using SV40 LT-specific monoclonal antibodies were performed from whole cell extracts: untreated (Cont) and etoposide treated (ETO) HEK293T cells (Input). The cell extracts (input) were then treated with Benzonase nuclease (see method). Immunoblotting of the Input (lanes 1, 2) and IP-LT (lanes 3, 4) were probed with antibodies to p53/TP53. The tumor protein p53 (p53/TP53) co-IPs with LT even after Benzonase treatment. The coIP of p53/TP53 without Benzonase treatment was observed in Lane 11 of Fig 2A and Lanes 3, 4 in Figure 9 in the main paper.

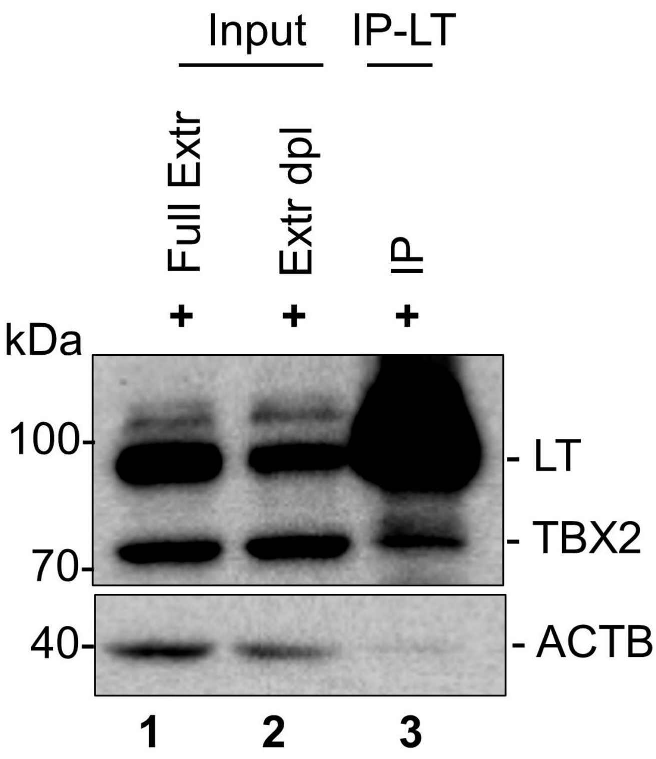

Figure S10: Co-immunoprecipitation of TBX2 (~ 72kDa) with SV40 LT after Benzonase treatment

Immunoprecipitation experiments using SV40 LT-specific monoclonal antibodies were performed from whole cell extracts of HEK293T cells (Input). The cell extracts (input) were then treated with Benzonase nuclease (see method). Immunoblotting of Input (Full Extr: before binding to antibody and Extr depl: after binding to antibody) (lanes 1, 2) and IP-LT (lanes 3) were probed with antibodies to SV40 LT and TBX2 (upper panel) and ACTB (β-actin lower panel). The TF, T-box transcription factor 2 (TBX2) (~72kDa) co-IPs with LT even after Benzonase treatment.
